# Utilizing prospective space-time scan statistics to discover the dynamics of coronavirus disease 2019 clusters in the State of São Paulo, Brazil

**DOI:** 10.1590/0037-8682-0607-2021

**Published:** 2022-08-05

**Authors:** Ricardo Vicente Ferreira, Marcos Roberto Martines, Rogério Hartung Toppa, Luiza Maria de Assunção, Michael Richard Desjardins, Eric Delmelle

**Affiliations:** 1 Universidade Federal do Triângulo Mineiro, Programa de Pós-graduação Stricto Sensu em Ciência e Tecnologia Ambiental, Uberaba, MG, Brasil.; 2 Universidade Federal de São Carlos, Centro de Ciências Humanas e Biológicas, Sorocaba, SP, Brasil.; 3 Universidade Federal de São Carlos, Departamento de Ciências Ambientais, Sorocaba, SP, Brasil.; 4 Universidade do Estado de Minas Gerais, Faculdade de Ciências Jurídicas, Ituiutaba, MG, Brasil.; 5 Department of Epidemiology, Spatial Science for Public Health Center, Johns Hopkins Bloomberg School of Public Health, Baltimore, MD, USA.; 6 University of North Carolina-Charlotte, Center for Applied Geographic Information Science, Department of Geography and Earth Sciences, Charlotte, NC, USA.

**Keywords:** Relative risk, Space-time statistics, SARS-CoV-2, Geographic information systems, Disease surveillance

## Abstract

**Background:**

The number of deaths and people infected with coronavirus disease 2019 (COVID-19) in Brazil has steadily increased in the first few months of the pandemic. Despite the underreporting of coronavirus cases by government agencies across the country, São Paulo has the highest rate among all Brazilian states.

**Methods:**

To identify the highest-risk municipalities during the initial outbreak, we utilized daily confirmed case data from official reports between February 25 and May 5, 2020, which were aggregated to the municipality level. A prospective space-time scan statistic was conducted to detect active clusters in three different time periods.

**Results:**

Our findings suggest that approximately 4.6 times more municipalities belong to a significant space-time cluster with a relative risk (RR) > 1 on May 5, 2020.

**Conclusions:**

Our study demonstrated the applicability of the space-time scan statistic for the detection of emerging clusters of COVID-19. In particular, we identified the clusters and RR of municipalities in the initial months of the pandemic, explaining the spatiotemporal patterns of COVID-19 transmission in the state of São Paulo. These results can be used to improve disease monitoring and facilitate targeted interventions.

## INTRODUCTION

Space-time surveillance of coronavirus disease 2019 (COVID-19) can be a powerful tool for mitigating the risk of transmission^1^ and can be useful for state and local health departments to monitor outbreaks in a timely manner^2^. In Brazil, the virus began to spread at the beginning of February 2020^3^; however, the Ministry of Health of Brazil officially reported that the first case occurred on February 25, 2020, in the state of São Paulo^4^. Large cities, such as São Paulo and Rio de Janeiro, were the main epicenters of the epidemic^5^. There were indications of rapid transmission within the state of São Paulo^6^ in municipalities with small populations and limited access to health care resources.

Monitoring the spread of COVID-19 is essential for the development of health strategies such as the allocation of tests and hospital beds. Prospective space-time scan statistics^7^ are powerful exploratory approaches for detecting active and emerging disease clusters. This approach determines whether the observed spatiotemporal patterns of the disease are randomly distributed or show a statistically significant cluster during the most recent period of analysis^8^.

In this study, we analyzed the initial outbreak of COVID-19 in the state of São Paulo, which exhibited the highest number of cases during the first months of the pandemic^9^. Using a space-time scan statistic, our study aimed to detect emerging COVID-19 clusters in municipalities in the state of São Paulo. We demonstrate the evolution of the relative risk (RR) and statistically significant clusters, considering three time periods across São Paulo municipalities.

## METHODS

On March 1, 2020, the Health Surveillance Secretary of the Ministry of Health of Brazil identified the first confirmed COVID-19 case by real-time reverse-transcriptase polymerase chain reaction (RT-PCR), following the protocol of the University Charité (Berlin, Germany). In Brazil, the notification system is decentralized, and municipalities can conduct tests in private or public laboratories. The results are then notified by the state health system, which then notifies the national government.

**FIGURE 1:**
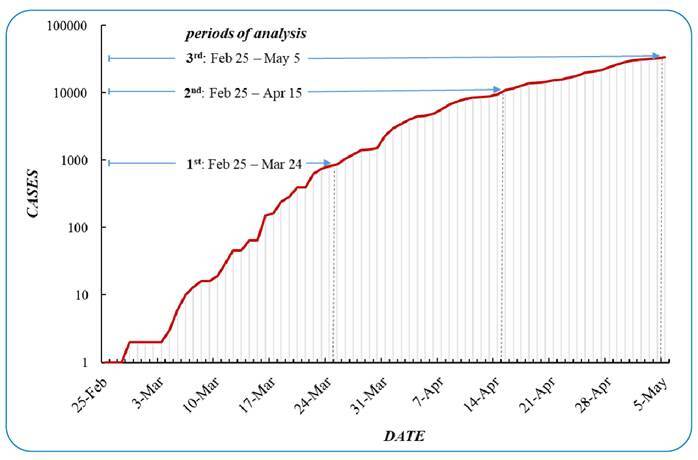
Cumulative number of coronavirus disease 2019 (COVID-19) cases in the state of São Paulo between February 25 and May 5, 2020 (logarithmic scale).

The state of São Paulo has approximately 46.6 million inhabitants in 2021 and an area of 248,219.5 square kilometers. On March 24, 2020, the governor of São Paulo mandated social distancing to prevent the spread of the novel coronavirus. As the incubation period for the novel COVID-19 is well supported by evidence to be approximately 14 days^10^, we selected three separate space-time analysis periods, from February 25 to March 24, February 25 to April 15, and February 25 to May 5, 2020, which included five incubation periods of onset of the most current COVID-19 case in the dataset (Figure 1).

The COVID-19 cases were retrieved from the Brazil.io project website^11^. This project compiles data from daily COVID-19 case reports by the municipality in the 27 Brazilian Federation Units and is available in a raw format, which has been organized in a format that SaTScan supports^12^. In the state of São Paulo, daily reports are prepared using the state data analysis system (https://www.seade.gov.br/coronavirus/) and contain the addresses of confirmed cases. The data include information on 645 municipalities in the state of São Paulo. In addition, we used records between February 25 and May 5, 2020, and a geographic information system (GIS) to geocode the locations^13^ of COVID-19 cases and aggregated them to the municipality level. Municipality-level GIS files were retrieved from the Instituto Brasileiro de Geografia e Estatística (IBGE).

Active clusters were detected using the Poisson space-time scan statistic method^8^, which was integrated into the SaTScan software. The prospective space-time statistic detects active clusters of disease, which is the excess incidence of cases during the last period of analysis^14^. The statistic systematically implements moving cylinders to scan the study area. The cylinders were centered on the centroid of the municipalities, the base of the cylinder was the spatial scanning window, and the height represented the temporal scanning window. The cylinders were expanded until the maximum spatial and temporal upper bounds were reached. We defined the upper bounds as having a maximum spatial and temporal scanning window size of 10% of the at-risk population to avoid extremely large clusters and 50% of the study period, respectively. In addition, each cluster's duration was set to a minimum of 2 days, and each cluster contained a minimum of five confirmed cases of COVID-19^15^.

 The Poisson model detects space-time clusters that were still occurring or active on the last day of the analysis^7^ ^,^ ^8^ ^,^ ^15^. We assumed that the data on COVID-19 cases in our study area followed the Poisson distribution. The null hypothesis states that the model reflects a constant risk, such as the absence of anomalous clustering of COVID-19. The alternative hypothesis states that the number of observed cases exceeds expected cases derived from the null model. The expected cases were calculated by multiplying the population in the cylinder by the total COVID-19 rate in the cylinder (*p*_*i*_ * C/P), with *p*_*i*_ the population in *i*; *C* the total COVID-19 cases in the state of São Paulo, and *P* the total estimated population in the state of São Paulo. We assumed that the study population was static during the study period. 

In SatSan, a maximum likelihood ratio test was used to identify cylinders with an elevated risk of contracting COVID-19. The cylinder has an elevated risk when the likelihood ratio is > 1, where the rate of cases inside the cylinder is greater than that outside the cylinder. To derive statistical significance, 999 Monte Carlo simulations were computed, and clusters were reported at the 95% confidence level (*P* < 0.05). Thus, 999 likelihood ratios were computed for each cylinder, where each cylinder was a potential cluster. To circumvent the assumption that the municipalities belonging to a cluster were homogenous, we also reported and mapped the RR for each municipality during each study period. RR was defined as the estimated risk of COVID-19 within a municipality divided by the risk outside the municipality (i.e., everywhere else).

## RESULTS

### First period of analysis: February 25 to March 24, 2020

**FIGURE 2:**
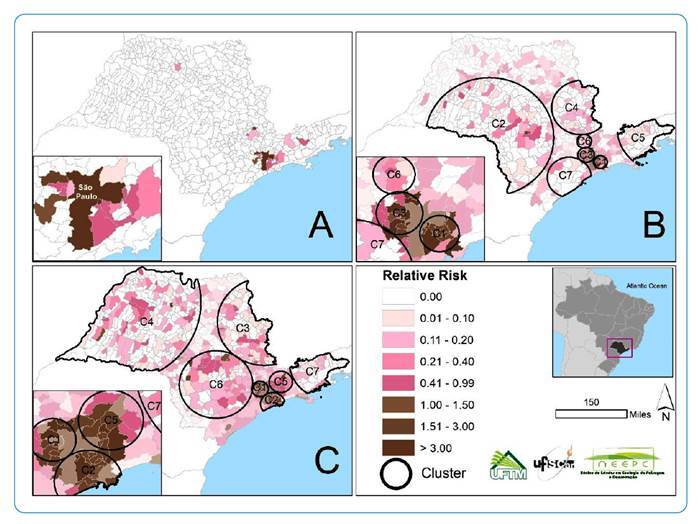
Spatial distribution of emerging space-time clusters of COVID-19 for the municipalities in the state of São Paulo, Brazil. **A:** First period of analysis: February 25 to March 24, 2020 (28 days). **B:** Second period of analysis: February 25 to April 15, 2020 (50 days; C1, RR = 3.29; C3, RR = 2.64; the other clusters did not show RR > 1). **C:** Third period of analysis: February 25 to May 5, 2020 (70 days; C1, RR = 3.71; C2, RR = 3.12; C5, RR = 2.68; the other clusters did not show RR > 1). **C1:** Cluster 1; **C2:** Cluster 2; **C3:** Cluster 3; **RR:** relative risk.

We did not detect any statistically significant emerging space-time clusters of COVID-19 between the first period of analysis (28 days) and two incubation periods (Figure 2A). However, we observed five municipalities with an RR > 1 outside the cluster, with more observed than that of the expected cases, for the first period. The highest RR was observed in São Paulo municipality (RR = 28.03), followed by São Caetano do Sul (RR = 3.42), Jaguariúna (RR = 2.38), Santana de Parnaíba (RR = 1.97), and Cotia (RR = 1.1) municipalities.

### Second period of analysis: February 25 to April 15, 2020

We detected seven statistically significant emerging space-time clusters of COVID-19 in the state of São Paulo for the second period of analysis (50 days, approximately three incubation periods), which included 353 municipalities. In the second period, there were two observed clusters with an RR > 1 (cluster 1: RR = 3.29; cluster 3: RR = 2.64), including 11 and 17 municipalities, respectively (Table 1). Both were located in the eastern region in the state of São Paulo, near São Paulo municipality (Figure 2B).

**TABLE 1:** Emerging space-time clusters of coronavirus disease 2019 (COVID-19) from February 25 to April 15, 2020, including relative risk (RR) estimates for the clusters identified in São Paulo, Brazil.

Cluster	Duration	Observed cases	Expected cases	RR	n of municipalities	Population	n of municipalities with RR > 1
1	3/30-4/15	943	305.05	3.3	11	3,845,800	4
2	3/22-4/15	118	522.04	0.2	203	4,475,331	2
3	3/31-4/15	641	252.45	2.6	17	3,381,536	6
4	3/22-4/15	86	421.67	0.2	61	3,614,906	0
5	3/22-4/15	22	168.99	0.1	33	1,448,732	0
6	3/22-4/15	36	127.66	0.3	9	1,094,410	0
7	3/22-4/15	21	96.37	0.2	19	826,174	0

*P*-value < .001; **n:** number; **RR:** relative risk.

Cluster 1 had 943 observed cases, with the highest RR in São Caetano do Sul municipality (RR = 2.23, with 85 observed cases for 38.34 expected cases), followed by Santos municipality (RR = 2.12, with 216 observed cases for 103.11 expected cases). Cluster 1 also included São Bernardo do Campo (RR = 1.16) and Santo André municipalities (RR = 1.09), with an RR > 1. Finally, cluster 3 contained 17 municipalities, including 6 with an RR > 1 and 641 observed cases (Table 1). The highest RR was in the Caieiras municipality (RR = 1.74), followed by Taboão da Serra (RR = 1.34), Santana de Parnaíba (RR = 1.24), Cotia (RR = 1.18), Franco da Rocha (RR = 1.12), and Osasco (RR = 1.04) municipalities.

### Third period of analysis: February 25 to May 5, 2020

We detected seven statistically significant emerging space-time clusters of COVID-19 in our third period of analysis (70 days, five incubation periods), covering 513 municipalities in the state of São Paulo. Clusters 1, 2, and 5 showed the highest RR (Table 2) with RR = 3.71, RR = 3.12, and RR = 2.68, respectively. All of these were located near São Paulo municipality in the eastern region of the state (Figure 2C).

**TABLE 2:** Emerging space-time clusters of COVID-19 from February 25 to May 5, 2020, including relative risk (RR) estimates for the clusters identified in São Paulo, Brazil.

Cluster	Duration	Observed cases	Expected cases	RR	n of municipalities	Population	n of municipalities with RR > 1
1	4/15-5/5	2,598	741.98	3.7	17	3,381,536	11
2	5/15-5/5	2,899	985.48	3.1	14	4,491,304	5
3	5/1-5/5	534	1,641.67	0.3	87	4,489,123	0
4	4/1-5/5	583	1,660.57	0.3	263	4,540,784	2
5	4/24-5/5	1,118	425.91	2.7	15	3,396,868	2
6	4/1-5/5	758	1,635.26	0.4	85	4,471,575	3
7	4/1-5/5	108	485.36	0.2	32	1,327,200	0

P-value < .001; **n:** number; **RR:** relative risk.

Cluster 1 included 11 municipalities with RR > 1, among which the highest RR was observed in the Barueri municipality (RR = 2.03). Cluster 2 had five municipalities with an RR > 1, among which the highest RR was in São Caetano do Sul municipality (RR = 1.84). Finally, cluster 5 had two municipalities with an RR > 1, in which the highest RR was in Igaratá municipality (RR = 1.84). All the previously mentioned municipalities border São Paulo municipality, including Barueri and São Caetano do Sul, except for Igaratá municipality, which is located 96 km away from São Paulo by road.

## DISCUSSION

In this study, we adapted the methodology used by Hohl et al.^16^, using three periods to detect emerging clusters of COVID-19 in the state of São Paulo, Brazil, utilizing confirmed case data from official reports. It is important to highlight that the RR throughout São Paulo increased during the three periods analyzed. In the first period (February 25 to March 24, 2020), within 28 days from the first recorded case, we detected only 5 municipalities with an RR > 1, whereas in the third period (February 25 to May 5, 2020), within 70 days from the first recorded case, we detected 23 municipalities with an RR > 1. These results show that within our study period, there were 4.6 times more municipalities with more observed than that of the expected cases in the state of São Paulo. Our results show that São Paulo municipality was a hotspot of COVID-19, which is highlighted by the location of the clusters with an RR > 1 in the second and third periods. However, 70 days after the spread of the virus in the state of São Paulo, we observed municipalities with an RR > 1 in almost all emerging clusters.

In the first period analyzed (February 25 to March 24, 2020), 20 municipalities had an RR for COVID-19. However, no clusters were detected, while all municipalities with reported COVID-19 incidence had a population greater than 50,000 inhabitants. In the second (February 25 to April 15, 2020) and third periods (February 25 to May 5, 2020), clusters with an RR > 1 were located around the municipality of São Paulo. However, in the second period, 25 municipalities with a population of less than 50,000 inhabitants began to exhibit COVID-19 incidence. In the third period, the number of municipalities with reported COVID-19 incidence increased to 117, demonstrating the spread to the countryside in the state^12^.

Regarding the limitations of this study, we acknowledge that there are inherent biases in the dataset we used, similar to other countries since tests were not always readily accessible when the pandemic arrived in Brazil, which led to the underreporting of cases and deaths. Another limitation is the determination of the SaTScan parameters^17^. The default configuration rarely produces useful and informative results because some clusters occupy a large proportion of the study area^18^. The task of determining the most suitable parameters is context-dependent and influenced by the geographic scale, processes that generate the clusters, and objectives of the study. 

This study presented an analysis of the space-time transmission dynamics of daily COVID-19 cases with the intent of identifying emerging space-time clusters active in the municipalities of the state of São Paulo in three different time periods. Three significant active clusters were detected in the state of São Paulo on May 5, 2020. Thus, this space-time approach is useful for infectious disease surveillance and identifying statistically significant clusters of cases and the RR of locations that belong to a cluster to facilitate further targeted interventions at finer spatial scales. We encourage researchers to utilize this approach for continued surveillance of COVID-19 worldwide, which can be strengthened by adjusting testing efforts and relevant covariates, such as poverty levels, access to healthcare, and vaccination rates.
